# *catena*-Poly[[diaqua­(1*H*-imidazo[4,5-*f*][1,10]phenanthroline)cobalt(II)]-μ-sulfato]

**DOI:** 10.1107/S1600536809016419

**Published:** 2009-05-07

**Authors:** Jian Yu

**Affiliations:** a Department of Chemistry, Lishui University, 323000 Lishui, Zhejiang, People’s Republic of China

## Abstract

The Co^II^ ion in the title complex, [Co(SO_4_)(C_13_H_8_N_4_)(H_2_O)_2_]_*n*_, has a slightly distorted octa­hedral coordination environment formed by two O atoms from two symmetry-related bridging sulfate ligands, two N atoms from a bis-chelating 1*H*-imidazo[4,5-*f*][1,10]phenanthroline (IPL) ligand and two O atoms from coordinated water mol­ecules. The bridging sulfate ligands connect Co^II^ ions to form a one-dimensional chain along the *b*-axis direction. In the crystal structure, inter­molecular O—H⋯O, O—H⋯N and N—H⋯O hydrogen bonds link the chains into a three-dimensional network.

## Related literature

For general background on coordination polymers, see: Ghosh *et al.* (2004 ▶). For related IPL coordination complexes, see: Xiong *et al.* (1999 ▶). For related structures of coordination polymers, see: Liu *et al.* (2008 ▶).
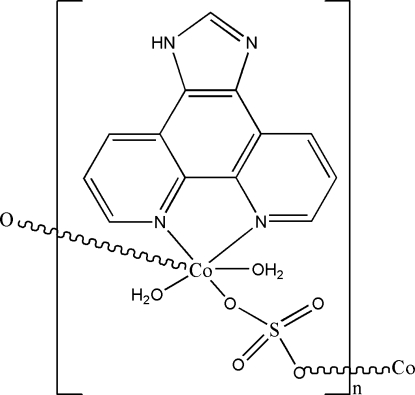


## Experimental

### 

#### Crystal data

[Co(SO_4_)(C_13_H_8_N_4_)(H_2_O)_2_]
*M* *_r_* = 411.26
Monoclinic, 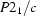

*a* = 10.916 (4) Å
*b* = 7.017 (2) Å
*c* = 19.690 (7) Å
β = 99.353 (7)°
*V* = 1488.2 (9) Å^3^
*Z* = 4
Mo *K*α radiation
μ = 1.34 mm^−1^
*T* = 298 K
0.27 × 0.15 × 0.10 mm

#### Data collection

Bruker APEXII area-detector diffractometer
Absorption correction: multi-scan (*SADABS*; Bruker, 2004 ▶) *T*_min_ = 0.714, *T*_max_ = 0.878
7263 measured reflections
2639 independent reflections
1216 reflections with *I* > 2σ(*I*)
*R*_int_ = 0.108

#### Refinement

*R*[*F* ^2^ > 2σ(*F* ^2^)] = 0.063
*wR*(*F* ^2^) = 0.100
*S* = 1.24
2639 reflections
242 parameters
16 restraints
H atoms treated by a mixture of independent and constrained refinement
Δρ_max_ = 0.53 e Å^−3^
Δρ_min_ = −0.80 e Å^−3^

### 

Data collection: *APEX2* (Bruker, 2004 ▶); cell refinement: *SAINT* (Bruker, 2004 ▶); data reduction: *SAINT*; program(s) used to solve structure: *SHELXS97* (Sheldrick, 2008 ▶); program(s) used to refine structure: *SHELXL97* (Sheldrick, 2008 ▶); molecular graphics: *SHELXTL* (Sheldrick, 2008 ▶) and *PLATON* (Spek, 2009 ▶); software used to prepare material for publication: *SHELXTL*.

## Supplementary Material

Crystal structure: contains datablocks I, global. DOI: 10.1107/S1600536809016419/lh2814sup1.cif

Structure factors: contains datablocks I. DOI: 10.1107/S1600536809016419/lh2814Isup2.hkl

Additional supplementary materials:  crystallographic information; 3D view; checkCIF report

## Figures and Tables

**Table 1:** Hydrogen-bond geometry (Å, °)

*D*—H⋯*A*	*D*—H	H⋯*A*	*D*⋯*A*	*D*—H⋯*A*
O2—H2*C*⋯O5	0.83 (5)	1.91 (3)	2.698 (7)	159 (7)
O1—H1*C*⋯O5^i^	0.82 (5)	2.00 (4)	2.749 (6)	150 (7)
O2—H2*B*⋯O6^ii^	0.82 (5)	2.18 (3)	2.957 (7)	158 (6)
O1—H1*B*⋯N4^iii^	0.84 (5)	1.91 (5)	2.731 (7)	168 (7)
N3—H3*A*⋯O4^iv^	0.86	1.95	2.795 (6)	168

Symmetry codes: (i) 
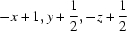
; (ii) 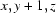; (iii) 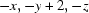; (iv) 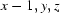.

**Fig. 1.**
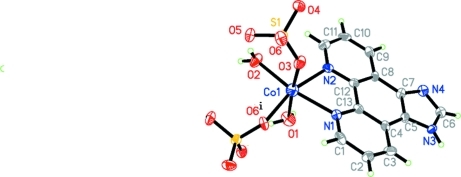
Molecular structure of the title compound showing the atom-labeling scheme [Symmetry codes: (i) -x+1,y+1/2,-z+1/2]. Displacement ellipsoids are shown at the 30% probability level.

**Fig. 2.**
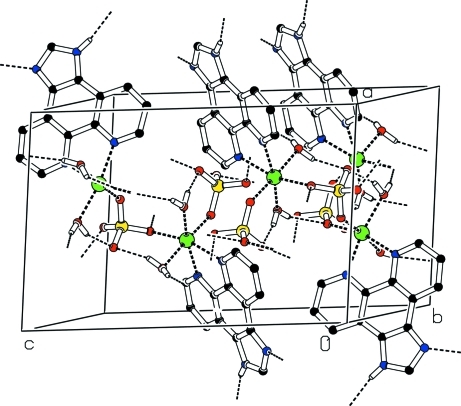
Part of the crystal structure showing hydrogen bonds as dashed lines.

## supplementary crystallographic information

### Comment

A wide range of extended one-dimensional, two-dimensional or three-dimenional frameworks with different interesting structural features, resulting from coordination bonding, hydrogen bonding, aromatic π···π stacking interactions play an important role in electron-transfer processes in some biological systems (Ghosh *et al.*,  2004). 1H-imidazo[4,5-f][1,10]-phenanthroline (IPL) is a rich conjugated molecular building block and has been employed to construct frameworks (Xiong *et al.*,  1999). Herein, we describe the preparation and crystal structure of the title complex (I).

The Co^II^ ion in the title complex, has a slightly distorted octahedral coordination formed by two O atoms from symmetry related bridging sulfate ligands, two N atoms from a bis-chelate IPL ligand and two O atoms from coordinated  water molecules (Figure 1). The Co—N and Co—O bond lengths are not significantly different from the values observed in related complexes (Liu *et al.*, 2008). The bridging sulfate ligands connect Co^II^ ions into a 1-D zigzag chain parallel to [0 1 0]. In the crystal structure, intermolecular O-H···O, O-H···N and N-H···O hydrogen bonds link one dimensional chains into a three dimensional network (Fig. 2). In addition, significant π···π stacking interactions with centroid to centroid distances in the range 3.465 (4)-3.548 (4)Å help stabilize the crystal structure.

### Experimental

The title compound was prepared by hydrothermal conditions. IPL (22mg, 0.1 mmol) in an aqueous solution (10 mL) was mixed with an aqueous solution (5mL) of Co(SO_4_)_2_ (31mg, 0.12mmol). After stirring for 30 min in air, the mixture was placed into 25 mL Teflon-lined autoclave and heated at 383K for 96h. The autoclave was cooled at a rate 5° h^-1^. The title complex as pink crystal was collected by filtration, washed with water, and dried in air.

### Refinement

All H atoms attached to C atoms and N atom were fixed geometrically and treated as riding with C—H = 0.93 Å; N-H = 0.86 Å with U_iso_(H) = 1.2U_eq_. H atoms of water molecule were located in difference Fourier maps and included in the subsequent refinement using restraints (O-H = 0.82 (1) Å).

### Crystal data

**Table:**

[Co(SO_4_)(C_13_H_8_N_4_)(H_2_O)_2_]	*F*(000) = 836
*M**_r_* = 411.26	*D*_x_ = 1.835 Mg m^−^^3^
Monoclinic, *P*2_1_/*c*	Mo *K*α radiation, λ = 0.71073 Å
Hall symbol: -P 2ybc	Cell parameters from 2639 reflections
*a* = 10.916 (4) Å	θ = 2.1–25.1°
*b* = 7.017 (2) Å	µ = 1.34 mm^−^^1^
*c* = 19.690 (7) Å	*T* = 298 K
β = 99.353 (7)°	Block, pink
*V* = 1488.2 (9) Å^3^	0.27 × 0.15 × 0.10 mm
*Z* = 4	

### Data collection

**Table:**

Bruker APEXII area-detector diffractometer	2639 independent reflections
Radiation source: fine-focus sealed tube	1216 reflections with *I* > 2σ(*I*)
graphite	*R*_int_ = 0.108
φ and ω scans	θ_max_ = 25.1°, θ_min_ = 2.1°
Absorption correction: multi-scan (*SADABS*; Bruker, 2004)	*h* = −13→11
*T*_min_ = 0.714, *T*_max_ = 0.878	*k* = −7→8
7263 measured reflections	*l* = −22→23

### Refinement

**Table:**

Refinement on *F*^2^	Primary atom site location: structure-invariant direct methods
Least-squares matrix: full	Secondary atom site location: difference Fourier map
*R*[*F*^2^ > 2σ(*F*^2^)] = 0.063	Hydrogen site location: inferred from neighbouring sites
*wR*(*F*^2^) = 0.100	H atoms treated by a mixture of independent and constrained refinement
*S* = 1.24	*w* = 1/[σ^2^(*F*_o_^2^) + 0.5747*P*] where *P* = (*F*_o_^2^ + 2*F*_c_^2^)/3
2639 reflections	(Δ/σ)_max_ < 0.001
242 parameters	Δρ_max_ = 0.53 e Å^−^^3^
16 restraints	Δρ_min_ = −0.80 e Å^−^^3^

### Special details

**Table:**

Geometry. All esds (except the esd in the dihedral angle between two l.s. planes) are estimated using the full covariance matrix. The cell esds are taken into account individually in the estimation of esds in distances, angles and torsion angles; correlations between esds in cell parameters are only used when they are defined by crystal symmetry. An approximate (isotropic) treatment of cell esds is used for estimating esds involving l.s. planes.
Refinement. Refinement of F^2^ against ALL reflections. The weighted R-factor wR and goodness of fit S are based on F^2^, conventional R-factors R are based on F, with F set to zero for negative F^2^. The threshold expression of F^2^ > 2sigma(F^2^) is used only for calculating R-factors(gt) etc. and is not relevant to the choice of reflections for refinement. R-factors based on F^2^ are statistically about twice as large as those based on F, and R- factors based on ALL data will be even larger.

### Fractional atomic coordinates and isotropic or equivalent isotropic displacement parameters (Å^2^)

**Table:**

	*x*	*y*	*z*	*U*_iso_*/*U*_eq_	
Co1	0.34886 (10)	0.82399 (16)	0.17466 (5)	0.0429 (4)	
S1	0.56896 (17)	0.4999 (3)	0.16741 (9)	0.0274 (5)	
N1	0.1702 (5)	0.6658 (8)	0.1762 (3)	0.0245 (14)	
N2	0.2484 (5)	0.8137 (8)	0.0647 (2)	0.0250 (14)	
N3	−0.2426 (5)	0.6042 (7)	0.0324 (3)	0.0267 (16)	
H3A	−0.2918	0.5566	0.0579	0.032*	
N4	−0.1771 (5)	0.7193 (8)	−0.0599 (2)	0.0291 (16)	
O1	0.2408 (5)	1.0847 (8)	0.1803 (2)	0.0445 (15)	
O2	0.5124 (5)	0.9751 (8)	0.1509 (3)	0.0412 (14)	
O3	0.4411 (4)	0.5535 (6)	0.1725 (2)	0.0350 (14)	
O4	0.5748 (4)	0.4383 (7)	0.09768 (19)	0.0386 (14)	
O5	0.6524 (4)	0.6627 (7)	0.18635 (19)	0.0323 (12)	
O6	0.6059 (4)	0.3399 (6)	0.21441 (19)	0.0307 (12)	
C1	0.1378 (7)	0.5856 (9)	0.2311 (3)	0.031 (2)	
H1A	0.1966	0.5749	0.2708	0.037*	
C2	0.0173 (7)	0.5157 (10)	0.2320 (3)	0.0332 (19)	
H2A	−0.0017	0.4581	0.2715	0.040*	
C3	−0.0704 (7)	0.5316 (10)	0.1759 (3)	0.034 (2)	
H3	−0.1503	0.4864	0.1764	0.041*	
C4	−0.0385 (6)	0.6200 (9)	0.1152 (3)	0.0238 (18)	
C5	−0.1175 (6)	0.6462 (10)	0.0526 (3)	0.0265 (18)	
C6	−0.2719 (6)	0.6509 (10)	−0.0334 (3)	0.0278 (18)	
H6	−0.3512	0.6371	−0.0585	0.033*	
C7	−0.0817 (6)	0.7170 (10)	−0.0066 (3)	0.0254 (18)	
C8	0.0452 (6)	0.7759 (9)	−0.0039 (3)	0.0216 (18)	
C9	0.0918 (7)	0.8457 (9)	−0.0621 (3)	0.0286 (18)	
H9	0.0403	0.8592	−0.1043	0.034*	
C10	0.2155 (7)	0.8930 (10)	−0.0542 (3)	0.036 (2)	
H10	0.2491	0.9375	−0.0917	0.043*	
C11	0.2904 (7)	0.8744 (9)	0.0098 (3)	0.032 (2)	
H11	0.3740	0.9063	0.0138	0.039*	
C12	0.1267 (6)	0.7589 (9)	0.0569 (3)	0.0226 (18)	
C13	0.0858 (6)	0.6824 (10)	0.1185 (3)	0.0237 (17)	
H1B	0.213 (6)	1.151 (9)	0.146 (2)	0.052 (12)*	
H2B	0.549 (6)	1.076 (5)	0.159 (3)	0.041 (7)*	
H1C	0.291 (5)	1.130 (10)	0.212 (2)	0.049 (9)*	
H2C	0.570 (4)	0.898 (7)	0.160 (3)	0.041 (7)*	

### Atomic displacement parameters (Å^2^)

**Table:**

	*U*^11^	*U*^22^	*U*^33^	*U*^12^	*U*^13^	*U*^23^
Co1	0.0385 (7)	0.0469 (8)	0.0425 (6)	0.0000 (7)	0.0044 (5)	−0.0012 (6)
S1	0.0247 (11)	0.0297 (13)	0.0272 (10)	0.0014 (11)	0.0024 (8)	−0.0027 (10)
N1	0.025 (4)	0.023 (4)	0.025 (3)	0.001 (3)	0.002 (3)	0.001 (3)
N2	0.028 (4)	0.023 (4)	0.025 (3)	0.002 (3)	0.008 (3)	−0.002 (3)
N3	0.020 (4)	0.037 (4)	0.025 (3)	−0.005 (3)	0.007 (3)	−0.001 (3)
N4	0.028 (4)	0.028 (4)	0.029 (3)	0.005 (3)	−0.001 (3)	0.004 (3)
O1	0.052 (4)	0.041 (4)	0.036 (4)	0.018 (3)	−0.008 (3)	0.007 (3)
O2	0.031 (4)	0.027 (4)	0.067 (4)	−0.004 (3)	0.013 (3)	0.006 (3)
O3	0.022 (3)	0.029 (4)	0.053 (3)	0.006 (3)	0.002 (3)	0.000 (2)
O4	0.040 (3)	0.054 (4)	0.023 (3)	−0.009 (3)	0.009 (3)	−0.005 (2)
O5	0.025 (3)	0.031 (3)	0.039 (3)	−0.005 (3)	−0.002 (2)	0.002 (3)
O6	0.035 (3)	0.026 (3)	0.027 (2)	0.004 (3)	−0.007 (2)	0.004 (2)
C1	0.042 (5)	0.027 (5)	0.020 (4)	0.001 (4)	−0.007 (4)	0.002 (3)
C2	0.036 (5)	0.035 (5)	0.031 (4)	−0.003 (5)	0.010 (4)	0.004 (4)
C3	0.033 (5)	0.042 (6)	0.029 (4)	0.005 (4)	0.012 (4)	−0.003 (4)
C4	0.025 (4)	0.011 (5)	0.034 (4)	0.003 (4)	0.003 (4)	−0.006 (3)
C5	0.023 (4)	0.025 (5)	0.031 (4)	−0.001 (4)	0.003 (4)	−0.006 (4)
C6	0.024 (4)	0.024 (5)	0.034 (4)	0.009 (4)	−0.002 (4)	−0.002 (4)
C7	0.022 (4)	0.021 (5)	0.032 (4)	0.001 (4)	0.002 (4)	0.004 (4)
C8	0.018 (4)	0.016 (5)	0.029 (4)	0.000 (3)	−0.001 (3)	−0.002 (3)
C9	0.035 (5)	0.018 (5)	0.032 (4)	0.002 (4)	0.001 (4)	−0.001 (4)
C10	0.051 (6)	0.036 (6)	0.028 (4)	−0.002 (4)	0.025 (4)	−0.002 (4)
C11	0.030 (5)	0.033 (5)	0.036 (5)	0.000 (4)	0.012 (4)	0.005 (4)
C12	0.023 (5)	0.021 (5)	0.024 (4)	0.005 (4)	0.005 (4)	0.000 (3)
C13	0.029 (4)	0.015 (4)	0.027 (4)	0.003 (4)	0.004 (3)	−0.003 (4)

### Geometric parameters (Å, °)

**Table:**

Co1—O3	2.152 (4)	O2—H2C	0.83 (5)
Co1—O6^i^	2.162 (4)	O6—Co1^ii^	2.162 (4)
Co1—O2	2.192 (5)	C1—C2	1.406 (9)
Co1—O1	2.190 (5)	C1—H1A	0.9300
Co1—N1	2.249 (5)	C2—C3	1.343 (8)
Co1—N2	2.263 (5)	C2—H2A	0.9300
S1—O4	1.451 (4)	C3—C4	1.438 (8)
S1—O3	1.465 (4)	C3—H3	0.9300
S1—O6	1.469 (4)	C4—C5	1.397 (8)
S1—O5	1.471 (5)	C4—C13	1.417 (9)
N1—C1	1.317 (7)	C5—C7	1.382 (8)
N1—C13	1.347 (7)	C6—H6	0.9300
N2—C11	1.312 (7)	C7—C8	1.438 (8)
N2—C12	1.368 (7)	C8—C12	1.375 (8)
N3—C6	1.325 (7)	C8—C9	1.414 (8)
N3—C5	1.391 (7)	C9—C10	1.374 (9)
N3—H3A	0.8600	C9—H9	0.9300
N4—C6	1.322 (7)	C10—C11	1.393 (9)
N4—C7	1.353 (8)	C10—H10	0.9300
O1—H1B	0.84 (5)	C11—H11	0.9300
O1—H1C	0.82 (5)	C12—C13	1.462 (8)
O2—H2B	0.82 (5)		
			
O3—Co1—O6^i^	91.96 (17)	N1—C1—C2	122.4 (7)
O3—Co1—O2	91.30 (19)	N1—C1—H1A	118.8
O6^i^—Co1—O2	97.5 (2)	C2—C1—H1A	118.8
O3—Co1—O1	174.6 (2)	C3—C2—C1	120.3 (7)
O6^i^—Co1—O1	86.69 (17)	C3—C2—H2A	119.8
O2—Co1—O1	94.1 (2)	C1—C2—H2A	119.8
O3—Co1—N1	88.53 (18)	C2—C3—C4	118.8 (7)
O6^i^—Co1—N1	93.88 (18)	C2—C3—H3	120.6
O2—Co1—N1	168.6 (2)	C4—C3—H3	120.6
O1—Co1—N1	86.3 (2)	C5—C4—C13	116.6 (6)
O3—Co1—N2	96.26 (18)	C5—C4—C3	126.3 (6)
O6^i^—Co1—N2	164.44 (18)	C13—C4—C3	117.0 (6)
O2—Co1—N2	95.5 (2)	C7—C5—N3	103.5 (6)
O1—Co1—N2	83.90 (18)	C7—C5—C4	125.0 (7)
N1—Co1—N2	73.20 (19)	N3—C5—C4	131.4 (6)
O4—S1—O3	109.3 (3)	N4—C6—N3	113.4 (6)
O4—S1—O6	108.6 (3)	N4—C6—H6	123.3
O3—S1—O6	108.7 (3)	N3—C6—H6	123.3
O4—S1—O5	110.5 (3)	N4—C7—C5	111.8 (6)
O3—S1—O5	109.9 (3)	N4—C7—C8	129.9 (6)
O6—S1—O5	109.8 (3)	C5—C7—C8	118.3 (7)
C1—N1—C13	119.4 (6)	C12—C8—C9	117.9 (6)
C1—N1—Co1	124.9 (5)	C12—C8—C7	119.4 (6)
C13—N1—Co1	114.9 (4)	C9—C8—C7	122.7 (6)
C11—N2—C12	117.5 (6)	C10—C9—C8	118.0 (6)
C11—N2—Co1	126.7 (5)	C10—C9—H9	121.0
C12—N2—Co1	115.3 (4)	C8—C9—H9	121.0
C6—N3—C5	107.3 (5)	C9—C10—C11	120.0 (6)
C6—N3—H3A	126.4	C9—C10—H10	120.0
C5—N3—H3A	126.4	C11—C10—H10	120.0
C6—N4—C7	104.1 (5)	N2—C11—C10	122.9 (7)
Co1—O1—H1B	124 (5)	N2—C11—H11	118.5
Co1—O1—H1C	94 (5)	C10—C11—H11	118.5
H1B—O1—H1C	121 (7)	N2—C12—C8	123.5 (6)
Co1—O2—H2B	140 (5)	N2—C12—C13	115.6 (6)
Co1—O2—H2C	105 (5)	C8—C12—C13	120.9 (6)
H2B—O2—H2C	101 (7)	N1—C13—C4	122.0 (6)
S1—O3—Co1	133.0 (3)	N1—C13—C12	118.2 (6)
S1—O6—Co1^ii^	132.0 (3)	C4—C13—C12	119.7 (6)
			
O3—Co1—N1—C1	79.0 (5)	C13—C4—C5—N3	−179.2 (7)
O6^i^—Co1—N1—C1	−12.9 (5)	C3—C4—C5—N3	2.5 (12)
O2—Co1—N1—C1	168.3 (9)	C7—N4—C6—N3	−0.6 (8)
O1—Co1—N1—C1	−99.3 (5)	C5—N3—C6—N4	0.7 (8)
N2—Co1—N1—C1	175.9 (6)	C6—N4—C7—C5	0.4 (8)
O3—Co1—N1—C13	−111.3 (5)	C6—N4—C7—C8	−179.5 (7)
O6^i^—Co1—N1—C13	156.9 (5)	N3—C5—C7—N4	0.0 (8)
O2—Co1—N1—C13	−22.0 (13)	C4—C5—C7—N4	177.6 (6)
O1—Co1—N1—C13	70.4 (5)	N3—C5—C7—C8	179.9 (6)
N2—Co1—N1—C13	−14.3 (4)	C4—C5—C7—C8	−2.5 (11)
O3—Co1—N2—C11	−88.4 (6)	N4—C7—C8—C12	−179.6 (7)
O6^i^—Co1—N2—C11	150.2 (7)	C5—C7—C8—C12	0.5 (10)
O2—Co1—N2—C11	3.5 (6)	N4—C7—C8—C9	−1.8 (12)
O1—Co1—N2—C11	97.1 (6)	C5—C7—C8—C9	178.3 (7)
N1—Co1—N2—C11	−174.9 (6)	C12—C8—C9—C10	−0.3 (10)
O3—Co1—N2—C12	99.8 (5)	C7—C8—C9—C10	−178.2 (6)
O6^i^—Co1—N2—C12	−21.7 (10)	C8—C9—C10—C11	−1.0 (10)
O2—Co1—N2—C12	−168.3 (5)	C12—N2—C11—C10	3.1 (10)
O1—Co1—N2—C12	−74.8 (5)	Co1—N2—C11—C10	−168.6 (5)
N1—Co1—N2—C12	13.2 (4)	C9—C10—C11—N2	−0.5 (11)
O4—S1—O3—Co1	−102.4 (4)	C11—N2—C12—C8	−4.4 (10)
O6—S1—O3—Co1	139.3 (3)	Co1—N2—C12—C8	168.2 (5)
O5—S1—O3—Co1	19.1 (4)	C11—N2—C12—C13	176.7 (6)
O6^i^—Co1—O3—S1	−89.7 (4)	Co1—N2—C12—C13	−10.7 (7)
O2—Co1—O3—S1	7.9 (4)	C9—C8—C12—N2	3.0 (10)
N1—Co1—O3—S1	176.5 (4)	C7—C8—C12—N2	−179.0 (6)
N2—Co1—O3—S1	103.6 (4)	C9—C8—C12—C13	−178.1 (6)
O4—S1—O6—Co1^ii^	168.3 (3)	C7—C8—C12—C13	−0.1 (10)
O3—S1—O6—Co1^ii^	−72.9 (4)	C1—N1—C13—C4	1.4 (10)
O5—S1—O6—Co1^ii^	47.3 (4)	Co1—N1—C13—C4	−168.9 (5)
C13—N1—C1—C2	0.1 (10)	C1—N1—C13—C12	−175.5 (6)
Co1—N1—C1—C2	169.4 (5)	Co1—N1—C13—C12	14.1 (8)
N1—C1—C2—C3	−1.0 (11)	C5—C4—C13—N1	179.6 (6)
C1—C2—C3—C4	0.5 (11)	C3—C4—C13—N1	−1.9 (10)
C2—C3—C4—C5	179.3 (7)	C5—C4—C13—C12	−3.5 (10)
C2—C3—C4—C13	0.9 (10)	C3—C4—C13—C12	175.0 (6)
C6—N3—C5—C7	−0.4 (7)	N2—C12—C13—N1	−2.3 (9)
C6—N3—C5—C4	−177.7 (7)	C8—C12—C13—N1	178.8 (6)
C13—C4—C5—C7	4.0 (11)	N2—C12—C13—C4	−179.3 (6)
C3—C4—C5—C7	−174.3 (7)	C8—C12—C13—C4	1.7 (10)

Symmetry codes: (i) −*x*+1, *y*+1/2, −*z*+1/2; (ii) −*x*+1, *y*−1/2, −*z*+1/2.

### Hydrogen-bond geometry (Å, °)

**Table:**

*D*—H···*A*	*D*—H	H···*A*	*D*···*A*	*D*—H···*A*
O2—H2C···O5	0.83 (5)	1.91 (3)	2.698 (7)	159 (7)
O1—H1C···O5^i^	0.82 (5)	2.00 (4)	2.749 (6)	150 (7)
O2—H2B···O6^iii^	0.82 (5)	2.18 (3)	2.957 (7)	158 (6)
O1—H1B···N4^iv^	0.84 (5)	1.91 (5)	2.731 (7)	168 (7)
N3—H3A···O4^v^	0.86	1.95	2.795 (6)	168

Symmetry codes: (i) −*x*+1, *y*+1/2, −*z*+1/2; (iii) *x*, *y*+1, *z*; (iv) −*x*, −*y*+2, −*z*; (v) *x*−1, *y*, *z*.
